# Comparison effects of *Ferula gummosa* essential oil and Beta-pinene Alginate nanoparticles on human melanoma and breast cancer cells proliferation and apoptotic index in short term normobaric hyperoxic model

**DOI:** 10.1186/s12906-023-04266-4

**Published:** 2023-11-28

**Authors:** Mahmoud Osanloo, Somayyeh Pishamad, Ali ghanbariasad, Elham Zarenezhad, Media Alipanah, Hiva Alipanah

**Affiliations:** 1 Department of Medical Nanotechnology, School of Advanced Technologies in Medicine, Fasa University of Medical Sciences, Fasa, Iran; 2 Student Research Committee, Fasa University of Medical Sciences, Fasa, Iran; 3 Department of Medical Biotechnology, School of Advanced Technologies in Medicine, Fasa University of Medical Sciences, Fasa, Iran; 4 Noncommunicable Diseases Research Center, Fasa University of Medical Sciences, Fasa, Iran; 5 Faculty of Veterinary Medicine, Razi University, Kermanshah, Iran; 6 Department of Physiology, School of Medicine, Fasa University of Medical Sciences, Fasa, Iran

**Keywords:** Neoplasm, Nanotechnology, Melanoma, Breast Cancer, Hyperoxia

## Abstract

**Background:**

Breast cancer is the most common cancer among women, and melanoma is the most dreadful type of skin cancer. Due to the side effects of chemotherapy drugs, the development of new herbal nano-medicines has been considered.

**Methods:**

This study first investigated the chemical composition of *Ferula gummosa* essential oil using GC-MS analysis; β-pinene, with 61.57%, was the major compound. Next, alginate nanoparticles containing β-pinene and the essential oil with particle sizes of 174 ± 7 and 137 ± 6 nm were prepared. Meanwhile, their zeta potentials were 12.4 ± 0.7 and 28.1 ± 1 mV. Besides, the successful loading of β-pinene and the essential oil in nanoparticles was confirmed using ATR-FTIR analysis. After that, their effects on viability and apoptotic index of human melanoma and breast cancer cells were investigated in normoxia and normobaric hyperoxia (NBO) conditions.

**Results:**

The best efficacy on A-375 and MDA-MB-231 cells was achieved by alginate nanoparticles containing the EO at hyperoxic and normoxia conditions; IC_50_ 76 and 104 µg/mL. Besides, it affected apoptosis-involved genes; as *Bax/Bcl-2* ratio was higher than 1, conditions for induction of apoptosis were obtained. Higher sensitivity was observed in the A-375 cell line treated with Alg-EO in the NBO model.

**Conclusions:**

Alginate nanoparticles containing *F. gummosa* EO could be considered for further investigation in anticancer studies. Also, it may be expected that NBO can be a new strategy for delaying cancer progression and improving nanotherapy efficacy.

## Introduction

Cancers, with 10 million deaths in 2020, were the leading cause of death worldwide; breast and skin cancer were among the five most common types [1]. Side effects of chemotherapeutic drugs have led researchers to develop new herbal drugs; essential oils (EOs) with anticancer effects are a proper alternative source [2]. EOs are naturally volatile oils secreted as secondary metabolites from different parts of aromatic plants such as roots, stems, leaves, and flowers. They also possess antioxidant, antibacterial, antitumor, and anti-leishmanial effects [3]. For instance, in the family of *Apiaceae, Ferula gummosa*, a perennial herb native to Iran and Turkmenistan, has been widely used in traditional medicine [4]. Its cytotoxic effects were reported on human malignant glioblastoma multiforme and oral squamous cell carcinoma cells [5, 6]. Interestingly, its gum induces apoptosis via the ROS mechanism in human leukemic cells [7]. Besides, its extracts inhibit angiogenesis in vitro [8]. Its anti-seizure, antibacterial, and insecticidal effects were also reported [9–11]. β-pinene is the major component of *F. gummosa* EO [12, 13]. Beta and alpha-pinene are well-known monoterpenes found in many EOs. They have various biological activities such as anticoagulant, antitumor, antimicrobial, antimalarial, antioxidant, anti-inflammatory, anti-leishmanial, and analgesic effects [14, 15].

Hypoxia, or the lack of difference between the amount of oxygen available and the percentage of oxygen utilization, usually occurs in cancer and ischemic heart diseases [16]. In cancer cells, the expression of genes with critical roles in angiogenesis, cell cycle, and metabolism is induced to adapt tumor cells to a hypoxic condition [17]. Hypoxia in the tumor is associated with increased tissue metastasis risk and decreased survival rate [18]. In addition, resistance to lack of oxygen as a pathophysiological property of solid tumors can reduce chemotherapy or radiation therapy efficacy and create a more malignant cancer phenotype [19]. In addition to in vitro studies, new research in animal and human models showed the different effects of hyperoxic treatment like hyperbaric hyperoxic (HBO) on burn injuries and tumor cells. In tissues suffering from wounds and burns, oxygen therapy causes faster tissue repair and saves cell death; oxygen enhancement in some cancer tissues has been associated with an increased apoptotic index of cancer cells [20, 21]. The dual behavior of non-cancerous and cancerous cells towards increased tissue oxygen is one of the most interesting biological phenomena. Won Kim et al. showed that normobaric hyperoxic (NBO) as another method of hyperoxic treatment did not show any significant hyperoxic damage in normal tissue and cells but suppressed the progression of lung cancer. In brief, they concluded that the therapeutic effect of NBO was probably due to the increase in reactive oxygen species activity and apoptosis [22]. Besides, NBO is non-invasive and easier to administer than HBO [22]. Therefore, it is possible to consider and evaluation the increase of oxygen content as an adjuvant and promising therapeutic method to reduce tumor hypoxia and resistance to chemotherapy or radiation therapy.

Nowadays, herbal medicine is also expanding among cancer patients to treat or reduce the toxicity of chemotherapy drugs as a complementary treatment method [23, 24]. Despite the biodegradability and biocompatibility of herbal anticancer compounds (such as EOs and major compounds), their effectiveness is lower than synthetic drugs, and their solubility in water should also be improved [25, 26]. Including them in nanoparticles is proposed as a promising approach for the stability, solubility, and efficacy improvement of (herbal) drugs [27, 28]. Alginate is an anionic, biocompatible, and biodegradable polymer widely used in drug delivery [29, 30].

To the authors’ best knowledge, no report was published on alginate nanoparticles containing *F. gummosa* EO and β-pinene. Besides, we evaluated the anticancer activity of nanoparticles containing *F. gummosa* EO and β-pinene on melanoma and breast cancer cells in the NBO model.

## Materials and methods

A-375 (ATCC: CRL-1619) and MDA-MB-231 (ATCC: HTB-26) were bought from the Pasteur Institute of Iran. *F. gummosa* EO was purchased from Pharmaceutical Company Essential Oil Dr. Soleimani. Sodium alginate, calcium chloride, and β-pinene were provided from Sigma Aldrich (USA).

**Fig. 1:**
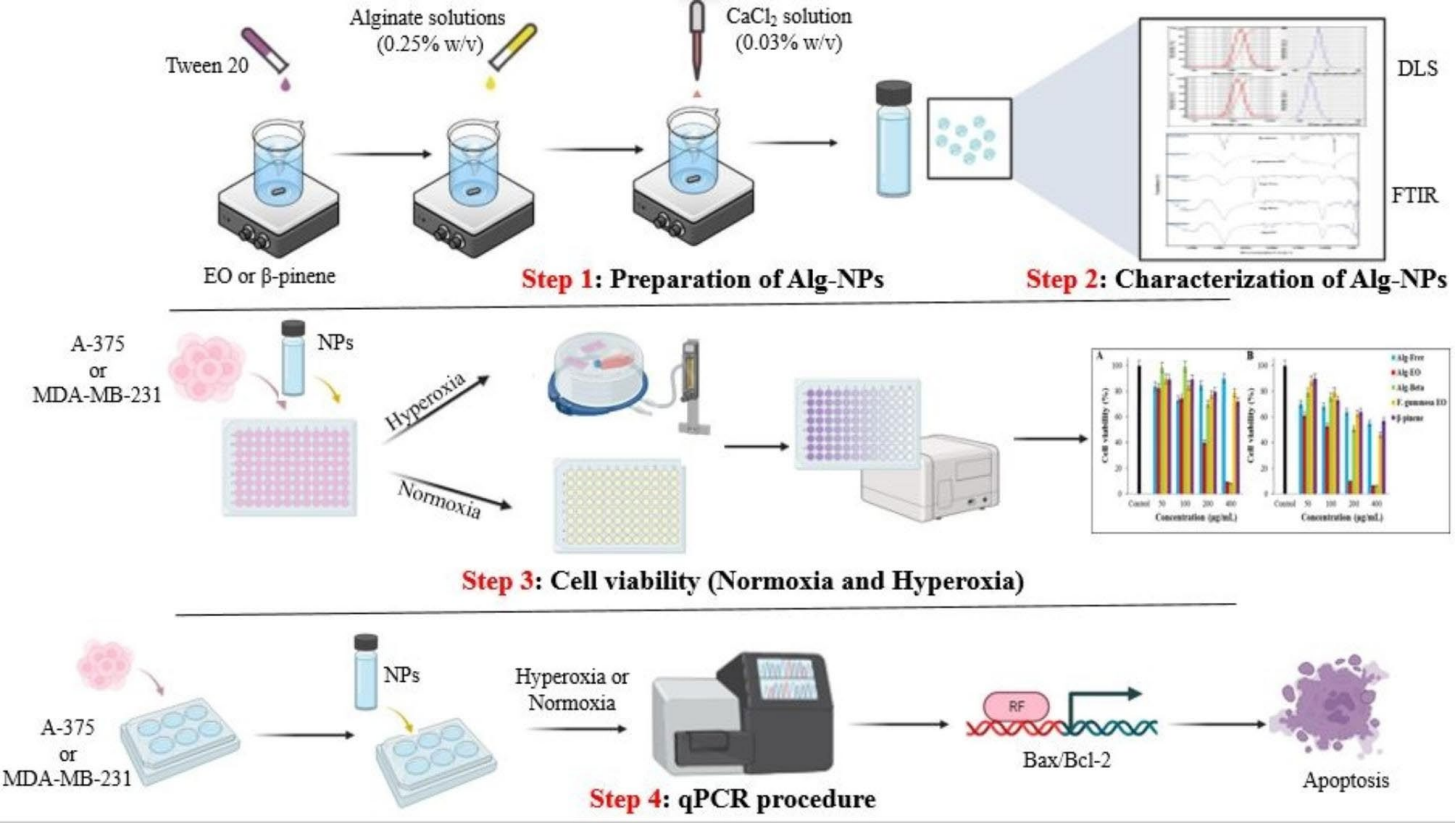
Schematic diagram of the experimental methods. EO: Essential oil, NPs: nanoparticles

### Identification of the chemical composition of *F. gummosa* EO

Identification of the chemical composition of *F. gummosa* EO was investigated using GC-MS analysis as described in our previous report [15]. The gas chromatography device used is Agilent 6890 with a column 30 m long, an Inner diameter of 0.25 μm, and a layer thickness of 0.25 μm of BPX5 type. To identify the essential oil’s components, the sample diluted by n-hexane was injected into the GC/MS machine in the amount of 1 µl. The column temperature program was adjusted as follows: The initial temperature of the oven is 50 °C and remaining at this temperature for 5 min, thermal gradient of 3 °C/minute, increasing the temperature up to 240 degrees Celsius and then at a rate of 15 degrees per minute, increasing the temperature up to 300 °C and kept for 3 min at this temperature and the response time was 75 min (Fig. 1). The temperature of the injection chamber (port) is 250 °C in a split from 1 to 35, and helium gas was used as a carrier gas with a flow rate of 0.5 ml/min. The mass spectrometer was the Agilent 5973 model with an ionization voltage of 70 electron volts, EI ionization method, and ionization source temperature of 220 °C. The scanning range of mass spectrometry was adjusted from 40 to 500. The software used was Chemstation. The spectra were identified with the inhibition index and compared with those found in reference books and articles using the mass spectra of standard compounds and the information available in the computer library [31, 32].

### Preparation and characterization of alginate nanoparticles containing *F. gummosa* EO and β-pinene

Ionic gelation was used to prepare alginate nanoparticles containing *F. gummosa* EO and β-pinene [33]. *F. gummosa* EO (0.16% w/v) or β-pinene (0.16% w/v) was first mixed with tween 20 (0.16% and 0.2% w/v, respectively) on magnetic stirrers (2000 rpm, RT), separately. Alginate solutions (0.25% w/v) were then added drop wisely and stirred for 5 min (2000 rpm, RT). After that, CaCl_2_ solution (0.03% w/v) was added and stirred for 40 min (2000 rpm, RT). Besides, free alginate nanoparticles were also prepared in the same approach without β-pinene. The prepared free alginate nanoparticles and those containing *F. gummosa* EO and β-pinene were abbreviated as Alg-Free, Alg-EO, and Alg-Beta (Step 1, Fig. 1).

Particle size, particle size distribution (SPAN), and zeta potential of the as-prepared samples were investigated using DLS type apparatus. SPAN is calculated as D90-D10/D50, where D is diameter and X are percentiles of nanoparticles with a lower diameter than these points. Moreover, ATR-FTIR analysis was used to confirm the loading of *F. gummosa* EO and β-pinene in nanoparticles. Spectra of *F. gummosa* EO, β-pinene, Alg-Free, Alg-EO, and Alg-Beta were recorded at 500–4000 cm^− 1^. The samples were subjected to the device without any modification.

Attenuated total reflectance-Fourier transform infrared (ATR-FTIR) spectroscopy is useful for determining functional groups in various compounds [34]. Analysis of ATR-FTIR (Bruker Company, Model Tensor II, USA) was used to confirm the presence of *F. gummosa* EO and β-pinene in the alginate nanoparticles; these spectra are recorded in the range of 400–4000 cm^− 1^(Step 2, Fig. 1).

### MTT assay procedure

MTT assay was used to investigate the cytotoxic effects of Alg-EO and Alg-Beta [35]. They were first diluted in PBS in serial dilutions of 50–800 µg/mL. The cells (A-375 and MDA-MB-231) were cultured in DMEM media culture containing 10% v/v FBS and 1% v/v penicillin/streptomycin. The cells were seeded (10^4^ cells/well) in 96-well plates and incubated at 37 °C, 21% O_2_, and 5% CO_2_ to reach 80 ± 5% confluence. Next, liquid media were replaced with 50 µL/well of fresh media culture. Afterward, by adding 50 µL/well of serial dilutions, the cytotoxic effects of Alg-EO and Alg-Beta were investigated at 50, 100, 200, and 400 µg/mL. Control group wells were treated with the used solvent (50 µL PBS), and the negative control group was treated with Alg-Free. Treated plates were incubated for 24 h at normal and hyperoxia conditions. Next, 50 µL/well of MTT solution (0.5 mg/m/L) was added, and plates were incubated for 4 h at normal and Hyperoxic conditions (The chamber was flushed with 60% oxygen at normal pressure). After that, liquid media was discarded, and 200 µL/well of DMSO was added to dissolve formazan crystals. OD of wells was read at 570 nm using a plate reader, and cell viability was calculated by OD sample / OD control × 100 (Step 3, Fig. 1).

The tests were performed three times, and the results are given as mean and standard deviations. IC_50_ values of samples were calculated using the free version of CalcuSyn.

### qPCR procedure

This study investigates the impact of β-pinene, *F. gummosa* EO, Alg-Beta, and Alg-EO on the expression of *Bax* and *Bcl-2* genes in A-375 and MDA-MB-231 cell lines using the qPCR technique [36]. Briefly, 50,000 cells per well were distributed onto 6-well plates and subjected to an overnight incubation period under the specified conditions. Subsequently, samples with obtained IC_50_ concentration was introduced into the designated wells and subjected to a 24-hour incubation period at normal and hyperoxia condition. The extraction of total RNAs from cells was performed using the Trizol RNA extraction Kit. Subsequently, an assessment was conducted to determine the quality and amount of the extracted RNA. This evaluation used the Nano-drop instrument (SYNERGY, HTX, multi-mode reader, BioTek Instruments, USA). The purity of the RNA and the presence of protein contamination were confirmed by ensuring that the OD ratio at 260 and 280 nm exceeded 1.8.

The synthesis of complementary DNA (cDNA) was carried out per the methods provided by the manufacturer. In summary, obtained total RNAs were combined with oligo dT and DEPC water and subjected to incubation at temperatures of 70 ºC and 4 ºC for 5 and 1-minute durations, respectively. Subsequently, a strand buffer with a concentration of 5X, deoxyribonucleotide triphosphates (dNTPs), ribonuclease inhibitor (RNasin), and Moloney Murine Leukemia Virus Reverse Transcriptase (M-MLV) were introduced. Subsequently, the microtubes underwent thermal cycling using a T100 thermocycler system manufactured by BIO-RAD in Germany. The thermal program was configured to run for a duration of 60 min at a temperature of 42 ºC. The resulting complementary DNAs (cDNAs) were then stored at -20 ºC.

The qPCR assay was conducted to examine the apoptotic regulatory genes *Bax* and *Bcl*-*2* and the expression of β-actin, which serves as a housekeeping gene. The primer sequences are provided in Table 1. To perform the amplification, a master mix containing forward and reverse primers, synthesized cDNA, and DEPC water (10, 0.5, 0.5, 1, and 8 µL, respectively) was prepared and transferred to a microtube. The microtube was then placed in an RT-PCR device (Step One Plus Real-Time PCR machine, AB Applied Biosystems, Germany). The normalization process employed Eq. 2^−ΔΔCT^, and the obtained data was presented as fold change relative to the control cells (Step 4, Fig. 1).

**Table 1:** Examined genes and their sequence

**β-actin**	Forward: 5′ - TCCTCCTGAGCGCAAGTAC − 3′ Reverse: 5′ - CCTGCTTGCTGATCCACATCT − 3′
**Bax**	Forward: 5′- CCCGAGAGGTCTTTTTCCGAG − 3′ Reverse: 5′ - CCAGCCCATGATGGTTCTGAT − 3′
**Bcl-2**	Forward: 5′ - GGTGGGGTCATGTGTGTGG − 3′ Reverse: 5′ - CGGTTCAGGTACTCAGTCATCC − 3′

### Statistical analysis

Statistical analysis of data (mean ± SD) was performed by one-way ANOVA using SPSS software with a confidence interval of 95%. Significance differences (p < 0.05) between the means evaluated by Tukey’s multiple comparison tests. The IC_50_ values were calculated using CalcuSyn software (Free version, BIOSOFT, UK).

## Results

### Chemical compositions of *F. gummosa* EO

Identified compounds in *F. gummosa* EO are listed in Table 2. β-pinene, 4.6-Guaiadinene, guaiol, bulnesol, and α –pinene with 61.57, 5.43, 5.28, 5.05, and 3.46% are five major compounds.

**Table 2:** Identified compounds in *F. gummosa* EO using GCMS analysis

NO	Retention Time	%	Components	Kovats Index	Type
1	10.93	0.16	α-thujene	930	NH
**2**	**11.31**	**3.46**	**α -pinene**	**939**	**NH**
3	12.19	0.14	camphene	954	NH
4	13.38	0.39	sabinene	975	NH
**5**	**13.69**	**61.57**	**β-pinene**	**979**	**NH**
6	14.21	2.38	myrcene	991	NH
**7**	15.19	1.25	δ -Caren	1002	NH
8	16.19	0.19	*para*-Cymene	1025	NH
9	16.34	0.56	limonene	1029	NH
10	16.45	0.38	β-phellandrene	1030	NH
11	16.68	0.73	α-ocimene	1037	NH
12	17.23	0.22	β-ocimene	1050	NH
13	23.79	0.32	1,3,5-undecatriene	1165	other
14	24.27	0.60	1,2,5-undecatriene	1185	other
15	25.07	0.22	myrtenol	1196	MO
16	29.06	0.27	bornyl acetate	1289	MO
17	31.88	0.78	terpinene-4-ol-acatate	1300	MO
18	32.77	0.1	cyclosativene	1371	SH
19	33.03	0.17	α-Copaene	1377	SH
20	33.63	0.09	β-Elemene	1391	SH
21	34.98	0.16	caryophyllene E	1419	SH
22	37.71	0.77	aromadendrene	1441	SH
23	38.32	0.13	α-muurolene	1500	SH
24	39.09	0.12	δ-cadinene	1523	SH
25	40.43	0.63	elemol	1550	SH
**26**	**42.33**	**5.28**	**guaiol**	**1601**	**SO**
27	44.40	0.2	asarone	1617	MO
28	44.70	0.8	β-Eudesmol	1651	SO
**29**	**44.99**	**5.43**	**4,6-Guaiadiene**	**1672**	**SO**
**30**	**45.71**	**5.05**	**bulnesol**	**1720**	**SH**
31	46.53	0.73	valencene	1768	SO

MH: Monoterpene Hydrocarbons, MO: Oxygenated monoterpenes, SH: Sesquiterpene Hydrocarbon, SO: Oxygenated sesquiterpene

### Particle size and zeta potential of Alg-EO and Alg-Beta

**Fig. 2:**
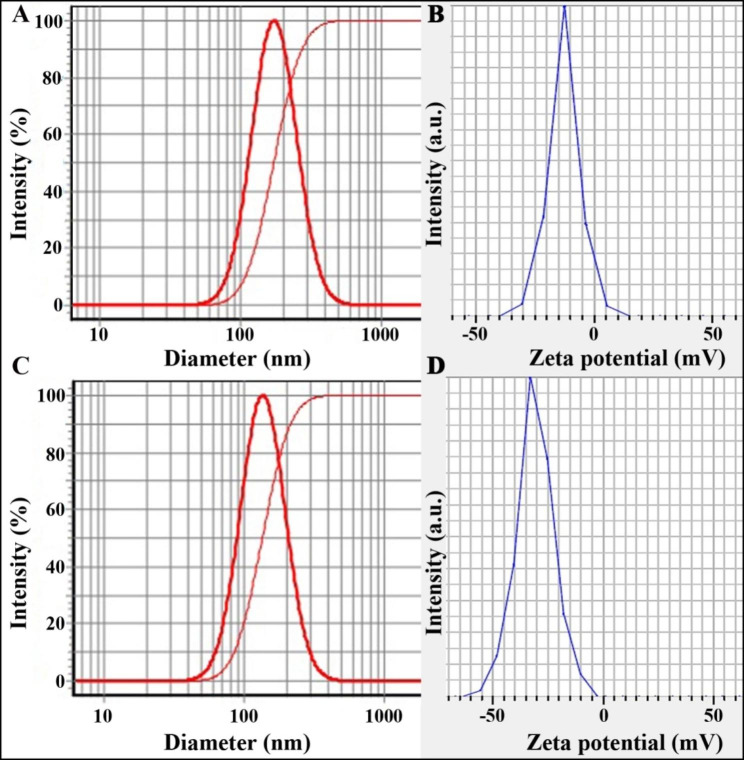
DLS and zeta potential diagrams of Alg-Beta: (**A**) particle size 174 ± 7 nm and (**B**) zeta potential 12.4 ± 0.7 mV and Alg-EO: (**C**) particle size 137 ± 6 nm and **D**) zeta potential 28.1 ± 1 mV

DLS and zeta potential diagrams of Alg-Beta with a particle size of 174 ± 7 nm, SPAN 0.94, and zeta potential 12.4 ± 0.7 mV are shown in Fig. 2A and B. Besides, these diagrams for Alg-EO are depicted in Fig. 2 C and D; particle size 137 ± 6 nm, SPAN 0.95, and zeta potential 28.1 ± 1 mV.

### Successful loading of β-pinene and *F. gummosa* EO in alginate nanoparticles

**Fig. 3:**
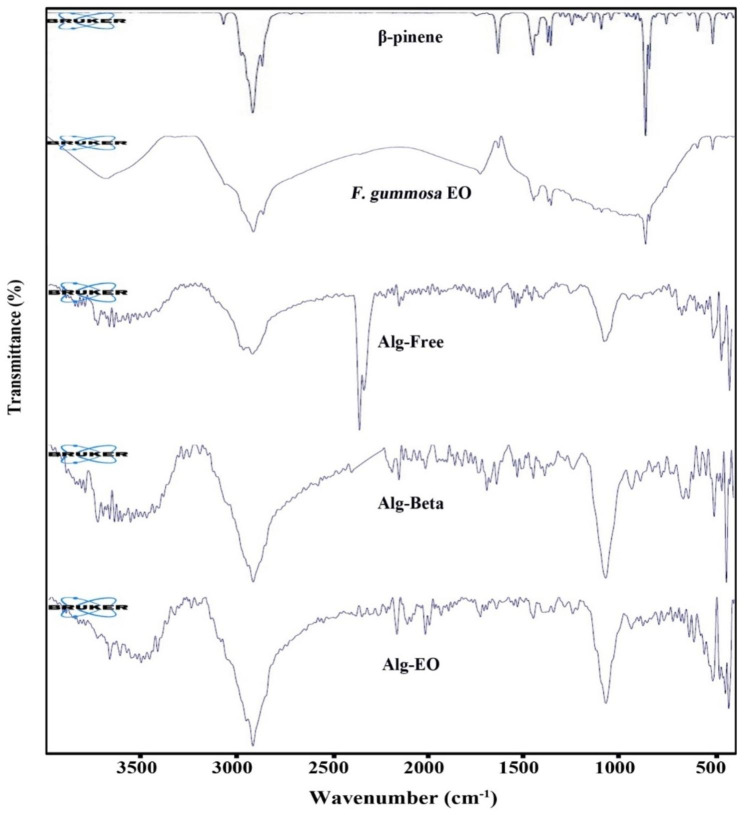
ATR-FTIR of samples. Alg-EO: alginate nanoparticles containing *F. gummosa* EO, Alg-Beta: alginate nanoparticles containing β-pinene

FTIR spectra of β-pinene showed that the bands at 3070 cm^− 1^ attributed to = C-H stretching vibration and that at 2978, 2918, and 2868 cm^− 1^ display –CH stretching vibration. The peak at 1640 and 1442 cm^− 1^ corresponds to the C = C skeleton vibration. The peak at 1053 cm^− 1^ is related to C-H bending absorption. The peak at 903 cm^− 1^ can be attributed to the vibration absorption of alkenes. The band at 717 cm^− 1^ is related to out-of-plane in C-H bending (Fig. 3).

ATR-FTIR spectrum of *F. gummosa* EO displayed the broad band between 3330 and 3690 cm^− 1^ attributed to the presence of OH group due to hydrogen bonding in the phenolic compound. The band at 2920 cm^− 1^ is attributed to C-H stretching. The peak at 1735 cm^− 1^ corresponded to C = O stretching that exhibited ester groups. The band at about 1457 cm^− 1^ is allocated to CH_2_ bending. The sharp band at 1103 cm^− 1^ is related to C-O stretching vibration. ATR-FTIR spectrum of blank showed the broad band between 3200 and 3600 cm^− 1^ are related to OH group due to hydrogen bonding in water and tween. The characteristic peak at 2922 cm^− 1^ displayed CH stretching vibration in tween, the bands at 2341 and 2555 cm^− 1^ can be related to CO_2_, and the peak at 1700 cm^− 1^ can be attributed to the carbonyl group in tween 20. The strong band at about 1076 cm^− 1^ can be related to C-O stretching in tween 20 (Fig. 3).

The ATIR spectra of alginate nanoparticles containing β-pinene displayed the broadband at about 3200–3700 cm^− 1^, related to OH groups, due to hydrogen bonding in water and tween, the band at 2923 cm^− 1^ can be attributed to C-H stretching vibration, the band at 1772 cm^− 1^ confirmed that the present of tween 20. The 1558 and 1351 cm^− 1^ peaks corresponded to carbonyl groups’ symmetric and asymmetric stretching vibration. The appearance of the other bands in the β-pinene and nanoparticles confirmed the successful loading of the β-pinene in the prepared nanoformulation.

The ATIR spectra of alginate nanoparticles containing *F. gummosa* EO showed a broad peak at about 3200–3700 cm^− 1^, attributed to hydroxyl groups, due to hydrogen bonding between EO, tween 20, and water, the band at 2959 cm^− 1^ can be related to C-H stretching vibration, the peak at 1734 cm^− 1^ demonstrated that the present of EO and tween 20. The bands at 1560 and 1352 cm^− 1^ can be attributed to carbonyl groups’ symmetric and asymmetric stretching vibration. The characteristic, strong peak at 1078 cm^− 1^ in alginate nanoparticles containing β-pinene and *F. gummosa* EO confirmed the reaction between the carboxyl group and Calcium ion (CO-Ca-CO group structure), which increases C-O vibration. This characteristic band showed ionic crosslinking. The appearance of the other bands in the β-pinene or EO and alginate nanoparticles containing them confirmed the successful loading of the EO and β-pinene in the prepared nanoformulation (Fig. 3).

### Cytotoxic effects of samples against A-375 and MDA-MB-231 cells

**Fig. 4:**
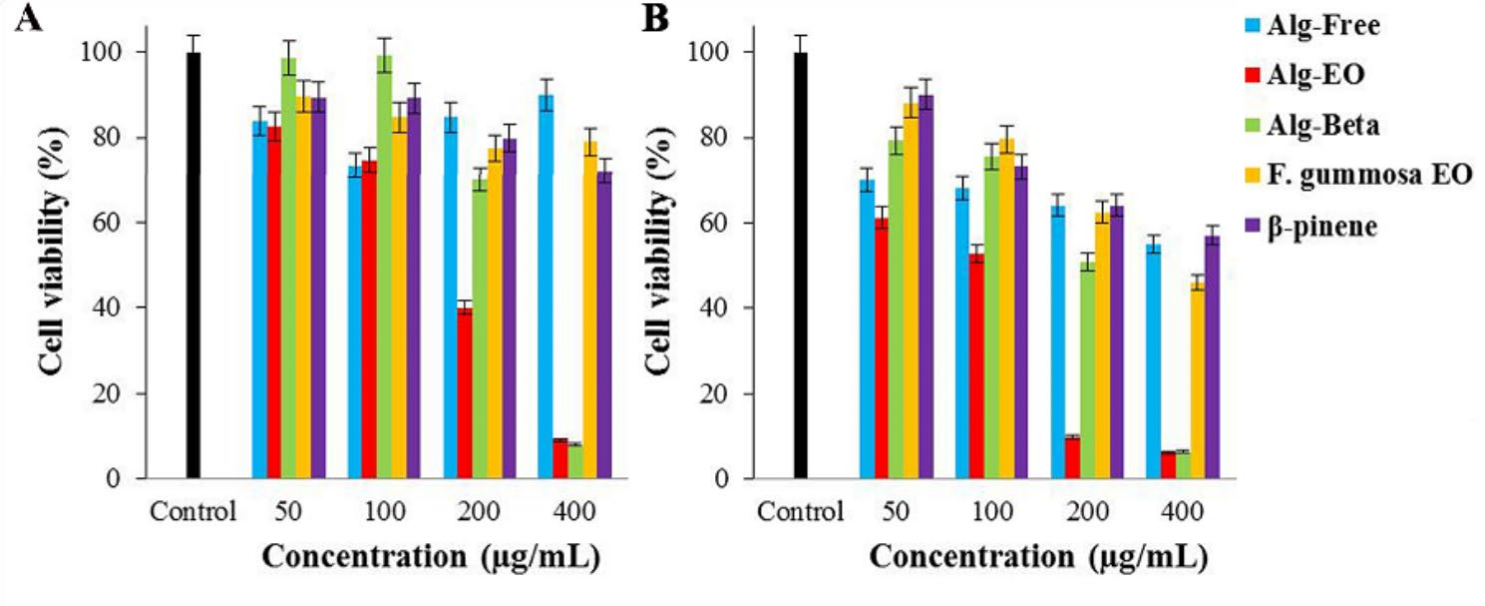
Cytotoxic effects of samples on A-375 cells; (**A**) Normoxia and (**B**) Hyperoxic condition

**Fig. 5:**
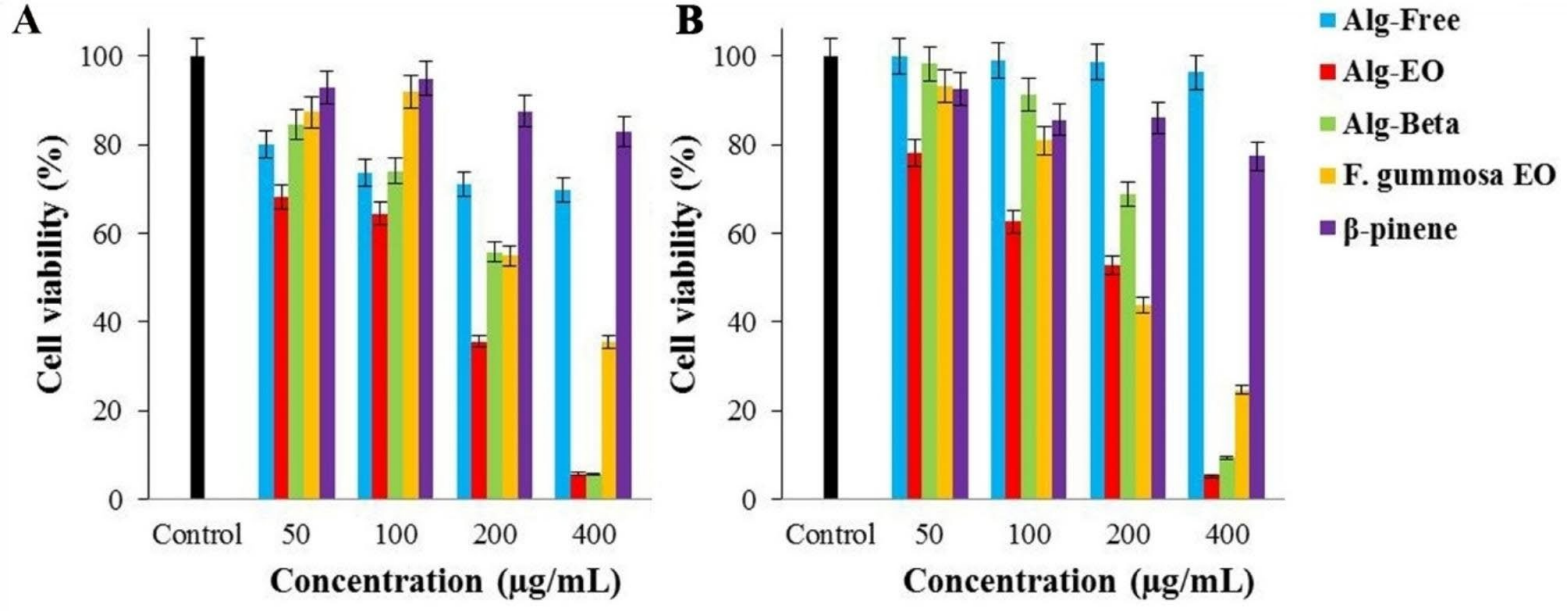
Cytotoxic effects of samples on MDA-MB-231 cells; (**A**) Normoxic and (**B**) Hyperoxic condition. Alg-EO: alginate nanoparticles containing *F. gummosa* EO, Alg-Beta: alginate nanoparticles containing β-pinene

Cytotoxic effects of β-pinene and *F. gummosa* EO and their nanoformulated states (Alg-Beta and Alg-EO) against A-375 and MDA-MB-231 cells at normoxic and hyperoxic conditions represented as cell viability (Figs. 4 and 5) and IC_50_ values (Table 3). As shown in Fig. 4, exposure of A-375 cancer cells with 200 µg/ml Alg-EO resulted in a substantial reduction (≈ 50%) in cell viability compared with the control group. Cell viability in Alg-Beta, *F. gummosa* EO, and β-pinene groups demonstrated almost similar values upon treatment at 200 µg/ml concentration. The results showed a dose-dependent manner in cancer A-375 cell proliferation upon treatment with Alg-Beta and Alg-EO. After 24h incubation with 400 µg/ml of Alg-Beta and Alg-EO, cell proliferation reduced to below 20% in normoxic and hyperoxic conditions. The highest cytotoxic effect appeared after incubation with Alg-EO in the NBO condition with IC_50_ = 76 µg/mL, where the IC_50_ value showed a 44% reduction compared to the normoxic condition (Table 3). As well as, treatment with the Alg-Beta, *F. gummosa* EO, and β-pinene in the NBO condition reduced IC_50_ values (≈ 46%, 14%, and 20%, respectively) compared with normoxic condition (Table 3).

As a result, at the end of 24h incubation, treatment with Alg-EO (IC_50_ = 104 µg/mL) and Alg-Beta (IC_50_ = 142 µg/mL) indicated relatively higher anticancer effect compared with their non-formulation (IC_50_ = 184 and 323 µg/mL, respectively) against MDA-MB-231 cells in normoxic condition (Fig. 5). In the NBO condition, regarding inhibition of cell proliferation, the cytotoxicity effects of Alg-EO and Alg-Beta were also more potent than their non-formulated states. It is important to note that the dose of Alg-beta (142 µg/mL) and β-pinene (323 µg/mL) to eradicate half population (50%) of MDA-MB-231 in normoxic was lower than in hyperoxic condition (205 and 536 µg/mL, respectively). Results in Table 3 also showed that both cancer cell lines were more sensitive to Alg-EO and Alg-beta than their non-formulation in normoxic and hyperoxic conditions.

**Table 3:** Obtained IC_50_ (µg/mL) values of samples against A-375 and MDA-MB-231 cells at normal and hyperoxic conditions

	A-375								
	Normoxia					Hyperoxia			
	Alg-EO	Alg-Beta	*F. gummosa* EO	β-pinene		Alg-EO	Alg-Beta	*F. gummosa* EO	β-pinene
**IC**_**50**_	136	248	262	302		76	132	226	242
	**MDA-MB-231**								
	**Normoxia**					**Hyperoxia**			
	**Alg-EO**	**Alg-Beta**	***F. gummosa*** **EO**	**β-pinene**		**Alg-EO**	**Alg-Beta**	***F. gummosa*** ** EO**	**β-pinene**
**IC**_**50**_	104	142	184	323		120	205	194	536

**Alg-EO**: alginate nanoparticles containing *F. gummosa* EO, **Alg-Beta**: alginate nanoparticles containing β-pinene

### Prediction of cancer cell sensitivity with *Bax/Bcl-2* ratio

**Fig. 6:**
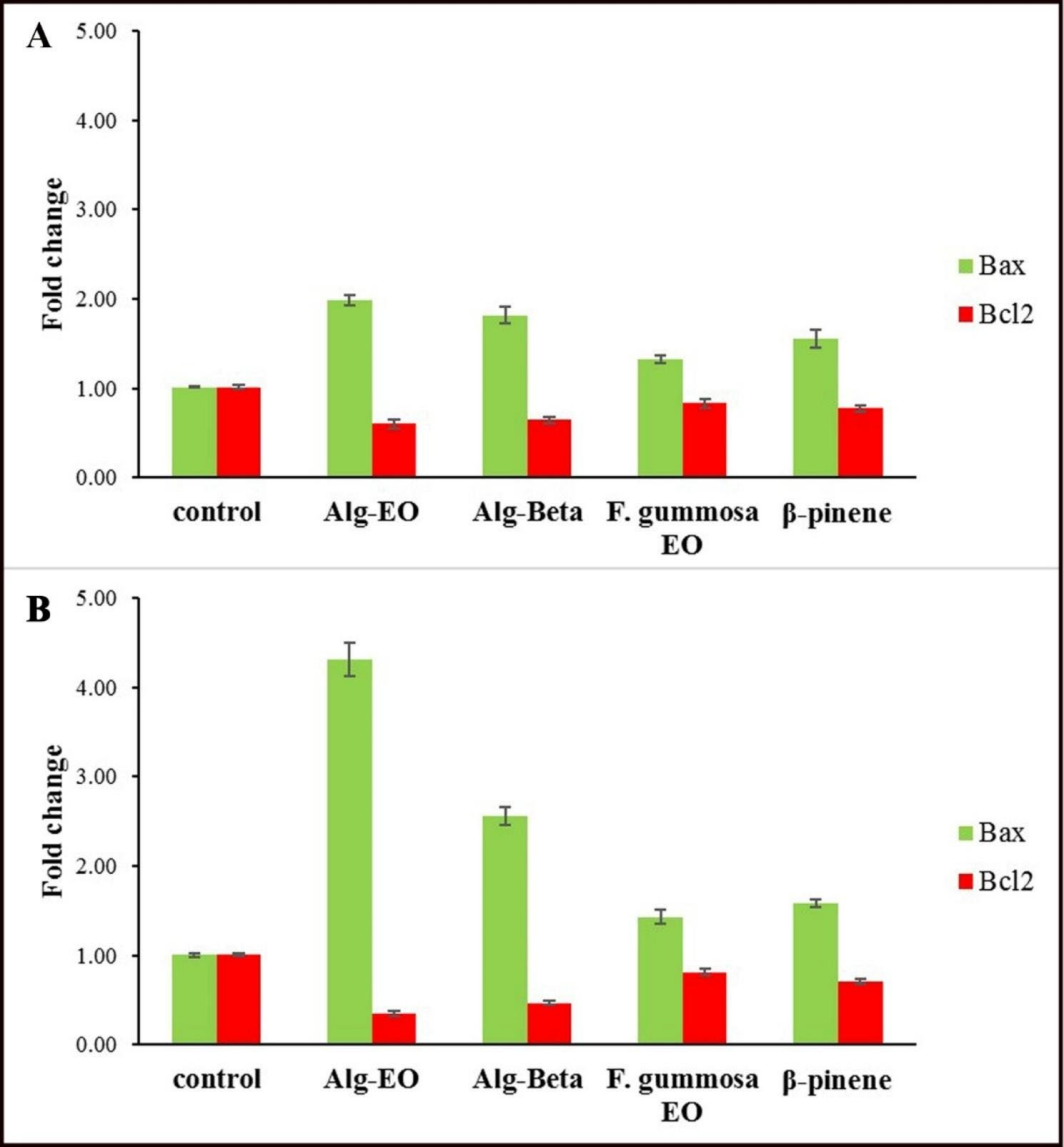
Effects of samples on *Bax* and *Bcl*-*2* genes expression in A-375 cells; (**A**) Normoxic and (**B**) Hyperoxic condition. Alg-EO: alginate nanoparticles containing *F. gummosa* EO, Alg-Beta: alginate nanoparticles containing β-pinene

**Fig. 7:**
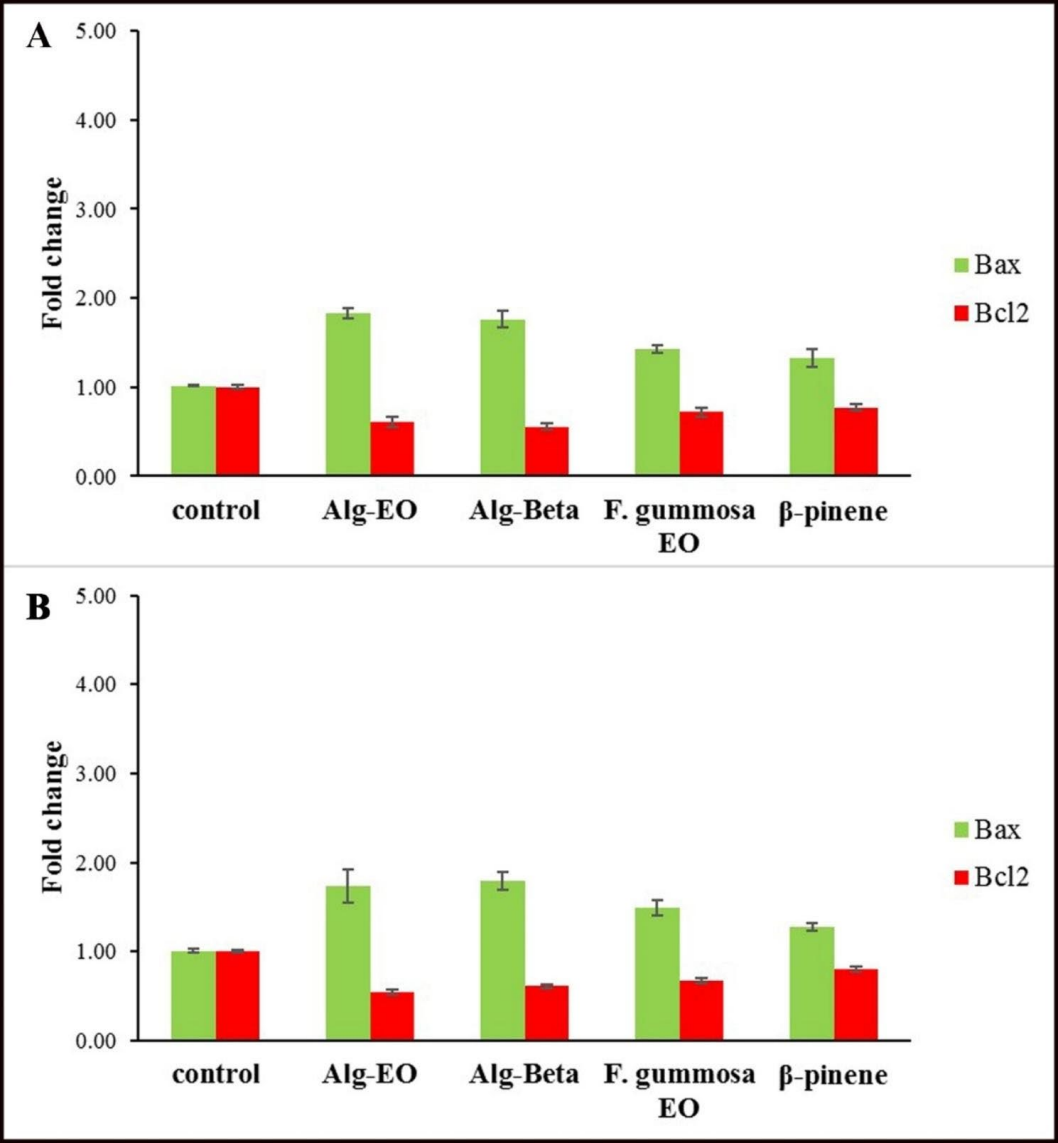
Effects of samples on *Bax* and *Bcl-2* genes expression in MDA-MB-231 cells; (**A**) Normoxic and (**B**) Hyperoxic condition. Alg-EO: alginate nanoparticles containing *F. gummosa* EO, Alg-Beta: alginate nanoparticles containing β-pinene

Effects of β-pinene, *F. gummosa* EO, Alg-Beta, and Alg-EO on *Bax* and *Bcl-2* gene expression in A-375 and MDA-MB-231 cells at normoxic and hyperoxic conditions are shown in Figs. 6 and 7. Cells were exposed to IC_50_ concentrations of the samples for 24 h and then analyzed for gene expression processing by qPCR (Figs. 6 and 7). Their *Bax/Bcl-2* ratio as an apoptotic index is summarized in Table 4. All samples at IC_50_ concentrations upregulated *Bax* (as a pro-apoptotic protein) in A-375 and MDA-MB-231 cancer cell lines in normoxic and hyperoxic models. The hyperoxic effect on overexpression of *Bax* in A-375 cells was more than normoxic condition. Also, a significant upregulation of *Bax* gene expression was observed in the Alg-EO and Alg-Beta nanoparticles groups compared to the control group. Our data also indicated that *Bcl-2* gene expression in A-375 cells was lower after treatment with Alg-EO and Alg-Beta in the NBO condition than normoxia. According to Table 4, Alg-EO and Alg-Beta increased apoptotic index (*Bax/Bcl-2* ratio) in the A-375 cancer cell line in normoxia, but this ratio was higher in the NBO model. Higher sensitivity was observed in the A-375 cell line treated with Alg-EO in hyperoxic condition. Examining the sensitivity of breast cancer cells to nanoparticles and hyperoxic showed that MDA-MB-231 cells are more sensitive to Alg-beta nanoparticles in normoxic and to Alg-EO nanoparticles in hyperoxic condition. The MDA-MB-231 cells showed the lowest sensitivity to β-pinene in normoxic and hyperoxic conditions.

**Table 4:** Correlation of cancer cell sensitivity to Alg-EO, Alg-Beta, *F. gummosa* EO, and β-pinene with *Bax/Bcl-2* ratio in normoxic and hyperoxic conditions

	A-375 cells							
	**Normoxic**				Hyperoxic			
**Sample**	*Bax*	*Bcl-2*	*Bax/Bcl-2* ratio	Susceptibility*	Bax	*Bcl-2*	*Bax/Bcl-2* ratio	Susceptibility*
Alg-EO	1.99	0.60	3.31	**+++**	4.31	0.35	12.44	++++
Alg-Beta	1.82	0.65	2.81	**++**	2.56	0.46	5.53	+++
EO	1.32	0.83	1.58	**+**	1.43	0.81	1.75	+
β-pinene	1.56	0.78	2.00	**+**	1.58	0.71	2.23	++
	MDA-MB-231 cells							
	**Normoxic**				Hyperoxic			
**SAMPLES**	*Bax*	*Bcl-2*	*Bax/Bcl-2* ratio	Susceptibility*	*Bax*	*Bcl-2*	*Bax/Bcl-2* ratio	Susceptibility*
Alg-EO	1.83	0.61	2.99	++	1.73	0.54	3.20	+++
Alg-Beta	1.76	0.56	3.17	+++	1.80	0.61	2.95	++
EO	1.43	0.72	2.00	+	1.49	0.67	2.24	++
β-pinene	1.33	0.77	1.72	+	1.27	0.80	1.59	+

**Alg-EO**: alginate nanoparticles containing *F. gummosa* EO, **Alg-Beta**: alginate nanoparticles containing β-pinene, **EO**: *F. gummosa* EO,

*A *Bax/Bcl-2* ratio < 1.00 characterizes cancer-resistant cells and a *Bax/Bcl-2* ratio > 1.00 cancer-sensitive cells.

+, low sensitivity (*Bax/Bcl-2* ratio 100–200% of control); ++, mild sensitivity (*Bax/Bcl-2* ratio 200-300% of control); +++, high sensitivity (*Bax/Bcl-2* ratio 300-600% of control), ++++, very high sensitivity (*Bax/Bcl-2* ratio > 600% of control).

## Discussion

EOs as herbal remedies have been widely used in traditional medicine for a long time. Several in vitro and in vivo studies have explored the antibacterial, antifungal, anti-inflammatory, and anticancer properties of EOs [2, 37, 38]. Nevertheless, knowing the biological activities of phyto-components such as EOs and improving their effectiveness is part of the main goals of cancer and complementary medicine researchers. At first, GC-MS was applied in our study to determine the chemical compositions of *F. gummosa* EO. Then, alginate nanoparticles containing *F. gummosa* EO and its main compound, i.e., β-pinene, were evaluated to investigate their anticancer properties in normoxic and hyperoxic conditions. Several studies have also reported the main ingredients of *F. gummosa* EO. For example, GC-MS analysis of *F. gummosa* fruits, as noted by Ghasemi et al., demonstrated β-pinene (43.78%) as the major component [39]. Ghannadi et al. also reported the main components of gum and latex of *F. gummosa* Boiss. grown in Iran was β-pinene (58.8%) [40]. Abbaszadegan et al. showed that β-pinene (51.83%) was the main compound of *F. gummosa* EO [41].

Laboratory and animal studies have shown that β-pinene has considerable antibacterial, antimalarial, antitumor, and gastro-protective effects. For example, Machado et al., by examining the anti-apoptotic effect of β-pinene against oral squamous cell carcinoma (OSCC), reported that β-pinene can be a possible new anticancer treatment for OSCC by inducing apoptotic index [42]. As well as a study was designed by Zhang et al. to investigate the combination of Paclitaxel and β–pinene against non-small-cell lung cancer cells and concluded that Paclitaxel and β–pinene have a synergistic anticancer effect [43]. In addition, in vitro analysis of the β-pinene anticancer effect on MCF-7 (breast cancer), A-375, and HepG2 (human hepatoma) cell lines done by Li et al. demonstrated antiproliferative activity of β-pinene against all cancer cell lines [44]. Alpha pinene is another terpenoid in *F. gummosa* EO with cancer-prevention properties. The therapeutic effect of α-pinene against prostate cancer (PC-3) growth was investigated by Zhao et al. in a xenograft model, which concluded that α-pinene had adverse toxicity on tumor progression and also induced cell cycle arrest and programmed cell death in PC-3 cell line [45]. Similarly, in a mice model, a fragrant environment enriched with α-pinene inhibited melanoma growth and reduced tumor volume by about 40% compared with the control group [46]. Inducing G2/M cell cycle arrest, inhibiting tumor invasion, and increasing the antitumor activity of natural killer cells are other anticancer mechanisms of alpha-pinene, which have been mentioned in the studies of Chen et al., Kang et al., and Jo et al. [43, 47].

Furthermore, *F. gummosa* EO can induce tumor cell death via multiple mechanisms like inducing apoptosis, inhibiting cell proliferation, and increasing cancer cells’ absorption of chemotherapy drugs. For example, flow cytometry and annexin-V analysis, as Gudarzi et al. noted, demonstrated that ethanolic extract of *F. gummosa* EO induced apoptosis and cell-cycle arrest in BHY cells (a human OSCC) [48]. These findings are congruent with results obtained by Gharaei et al., who reported that ethanol extracts of *F. gummosa* EO inhibited the cell proliferation of the human gastric cancer cell line, AGS, in a dose-dependent manner by inducing an apoptosis pathway [49]. Afshari et al. also evaluated the cytotoxicity of *F. gummosa* gum against U87 glioblastoma cells. Data indicated apoptogenic impacts of *F. gummosa* gum by upregulating of *Bax/Bcl-2* ratio [50]. Forouzmand et al. have explored the anticancer effects of *F. gummosa* in a radiation toxicity study against cervical cancer (Hela) cells and hypothesized that *F. gummosa* as a radiosensitizer agent had a synergistic effect with radiotherapy for inducing apoptosis in Hela cell [51]. Our in-vitro examination ensured *F. gummosa* EO and β–pinene anticancer impact, which was confirmed by the apoptotic index. Treatment with β-pinene showed a weak anticancer effect on A-375 (IC_50_ = 302 µg/mL) and MDA-MB-231 (IC_50_ = 323 µg/mL) cancer cells in normoxia condition, compared with *F. gummosa* EO (IC_50_ = 262 µg/mL and 184 µg/mL respectively). Because the nanoformulation of herbal medicines can increase their biological and pharmacological activities, we also used in vitro cancer models to evaluate AlgNPs containing *F. gummosa* EO and β–pinene. In vitro treatment of melanoma and breast cancer cells by AlgNPs markedly reduced cell viability percentages in cancer cells. Obtained IC_50_ for AlgNPs containing *F. gummosa* EO and β-pinene was significantly lower than non-formulation forms in both normoxia and hyperoxia conditions. Both cancer cell lines were the most sensitive to the anticancer effect of AlgNPs containing *F. gummosa* EO compared to Alg-Beta. From the literature, nanoemulsion from *F. gummosa* EO, as noted by Nosrat et al., can also reduce cell growth in the colon cancer model [52]. In addition, treatment with chitosan-*F. gummosa* EO nanocomposite, as speculated by Valinezhad et al., showed stronger antibacterial activities [53]. The nanoformulation as a feasible strategy for improving anticancer properties of plant EOs is confirmed by various studies [54–56].

In addition, it has been well established that the presence of a hypoxic environment in solid tumors can increase the aggressive characteristics of cancer tumors by increasing the expression of various genes such as HIF-1 (hypoxia-inducible factor 1), VEGF (vascular endothelial growth factor), EGF (epidermal growth factor) and IGF-2 (insulin-like growth factor 2), followed by increased angiogenesis and tissue metastasis [57, 58]. Meanwhile, hypoxia can reduce the effectiveness of standard cancer therapy, such as chemotherapy and radiotherapy [57]. Oxygen therapy using NBO and HBO has been proposed as a therapeutic strategy to reverse tumor hypoxia. The anticancer activity of hyperoxia has also been shown in laboratory and animal models; of course, the NBO method has received more attention than HBO because of its ease and fewer complications. Accordingly, our study evaluated the synergic effect of short-term NBO on the anticancer activity of Alg-EO and Ag-Beta against breast and melanoma cancer cells. Nanotherapy (Alg-EO and Alg-Beta) performed under NBO induced apoptosis and caused a considerable reduction in cell viability of A-375 cells with lower IC_50_ (76 and 132 µg/mL) compared with normoxia condition (136 and 248 µg/mL). It has been shown that hyperoxia with 60% oxygen can prevent the survival and proliferation of cancer cells by increasing oxidative stress and inducing apoptosis, and restoring the sensitivity to chemotherapy agents can provide the conditions for tumor regression [22, 59, 60]. This data is congruent with results obtained by Lee et al., who demonstrated hyperoxia re-sensitized human glioblastoma multiforme cells (D54 and U87) to temozolomide [61]. Similar to our results, the NBO condition, as noted by Kim et al., induced oxygen toxicity against the progression of lung cancer by inducing oxidative stress and apoptosis pathways [22]. Moon et al. showed that intermittent NBO alone or combined with carboplatin could be tumoricidal by induction of oxidative stress and apoptosis [62]. It has also been shown that, hyperoxia up to 60% O_2_ increased lactate and intracellular acidification and significantly retarding tumor growth and metastasis in lung cancer [63]. In addition, Bels et al. also examined hyperoxia up to 60% O_2_ against hematopoietic lymphoid cancer cell lines and had adverse inhibitory effects on leukemia cell proliferation [64]. Contrariwise, Tiron et al. indicated that the metastasis rate following long-term hyperoxia (80% O_2_) increased in the 4T1 triple-negative breast cancer model [65]. Our examination also assured that Alg-EO and Alg-Beta enhanced MDA-MB-231 breast cancer cell growth arrest, but hyperoxia (60% O_2_) did not enhance this impact.

On the other hand, most of the previous studies on oxygen therapy have focused more on hyperbaric hyperoxia. For example, hyperbaric oxygen treatment, as noted by Chen et al., unlike non-hematopoietic (A549, MCF-7) cells, can induce apoptosis in hematopoietic (Jurkat, NCI-H929) cells [66]. Similarly, Granowitz et al. showed that HBO caused no increase in apoptotic index in the MCF7 human mammary adenocarcinoma cell line [67]. Moreover, Sun et al. also investigated the effect of HBO on human oral cancer and concluded that apoptosis showed no change after HBO therapy [68]. Hjelde et al. also investigated hyperoxia (400 kPa O_2_) synergic effect on photodynamic therapy (PDT) on human colon carcinoma (SW480 and WiDr) and rat bladder cell carcinoma (AY-27) cell lines. They found that hyperbaric oxygen had no synergic effect on the photo-killing impact of PDT [69]. Following these findings, in most experimental and clinical studies, improved PDT outcomes have been established in hyperbaric oxygen-treated groups [70–72]. In addition, Kawasoe et al. reported that hyperbaric oxygen suppressed both tumor volume and number by enhancing carboplatin’s chemotherapeutic effects in the osteosarcoma mice model [73]. Our data indicated that nanotherapy with Alg-EO and Alg-Beta modulate and block the cell viability in melanoma and breast cancer. Unlike the triple-negative breast cancer cell line (MDA-MB-231), normobaric hyperoxia in melanoma cells increased the anticancer effects of nanoparticles, which could be confirmed by increasing the apoptotic index. This disagreement may be due to different thresholds for apoptosis in cancer cells with different origins [66]. On the other hand, the design and different hyperoxia conditions, duration of hyperoxia, and oxygen pressure can be the reasons for the differences observed in the mentioned studies. In addition, a more detailed investigation of apoptosis with live and dead staining (LDA) images and Ki-67 proliferation marker, IF staining along with DAPI and F-actin and also measuring the expression of different caspases can be helpful in discovering how cancer cells getting apoptotic following treatment with Alg-EO and Alg-beta.

## Conclusion

The study evaluated the anticancer potential of alginate nanoparticles containing *Ferula gummosa* EO and β-pinene against melanoma and breast cancer cells under normoxic and hyperoxic conditions. Alg-EO and Alg-Beta induced apoptosis in melanoma and breast cancer cells confirmed by increased *Bax/Bcl-2 ratio*. Our findings provide the first evidence that combination therapy with NBO enhanced apoptotic index in Alg nanoparticle-exposed melanoma cells, and it may be expected that NBO can be an alternative for cancer treatment. The study highlights the potential of combining natural herbal compounds with nanotechnology to improve their therapeutic efficacy, especially in conjunction with hyperoxic for cancer treatment.

## Data Availability

The datasets used and/or analysed during the current study available from the corresponding author on reasonable request.

## Declarations

### Research involving humans and animals statement

This research did not involve human and animal studies.

## Abbreviations

EO: Essential oil
Alg-EO: Alginate nanoparticles containing *F. gummosa* EO
Alg-Free: Free Alginate nanoparticles
Alg-Beta: Alginate nanoparticles containing β-pinene
NBO: Normobaric Hyperoxic
HBO: Hyperbaric Hyperoxic
